# Reduction of 4-Nitrophenol to 4-Aminophenol by Reusable CuFe_5_O_8_-Based Catalysts Synthesized by Co-Precipitation Method

**DOI:** 10.3390/molecules30040777

**Published:** 2025-02-07

**Authors:** Patompong Siri-apai, Sila Yaemphutchong, Natapol Suetrong, Arunthip Suesuwan, Nicha Choophun, Suttipong Wannapaiboon, Aphichart Rodchanarowan, Kantapat Chansaenpak, Nidcha Aroonrote, Yuranan Hanlumyuang, Worawat Wattanathana

**Affiliations:** 1 Department of Materials Engineering, Faculty of Engineering, Kasetsart University, Ladyao, Chatuchak, Bangkok 10900, Thailand; patompong.s@ku.th (P.S.-a.); sila.ya@ku.th (S.Y.); natapol.s@ku.th (N.S.); arunthip.s@ku.th (A.S.); nicha.choo@ku.th (N.C.); fengacrw@ku.ac.th (A.R.); 2 Synchrotron Light Research Institute, Suranaree, Muang, Nakhon Ratchasima 30000, Thailand; suttipong@slri.or.th; 3 National Nanotechnology Center, National Science and Technology Development Agency, Thailand Science Park, Pathum Thani 12120, Thailand; kantapat.cha@nanotec.or.th; 4 Center for Advanced Studies in Nanotechnology for Chemical, Food and Agricultural Industries, Kasetsart University, Bangkok 10900, Thailand; nidcha.a@ku.ac.th

**Keywords:** copper ferrite, co-precipitation, reduction, 4-nitrophenol, reusable catalyst

## Abstract

The reduction of unfriendly 4-nitrophenol to make it unimpactful with the environment (4-aminophenol) was carried out using the metastable form of copper ferrite (CuFe_5_O_8_) synthesized by the co-precipitation of metal nitrate salts, an efficient method with inexpensive and abundant starting materials. The samples were obtained by calcination at various temperatures ranging from 600 °C to 900 °C. The material characterizations, including X-ray diffraction, N_2_ adsorption/desorption, scanning electron microscope, X-ray absorption spectroscopy, and ultraviolet–visible spectrometry, were employed to identify the detailed structures and describe their correlations with catalytic activities. The X-ray diffraction and X-ray absorption spectroscopy analyses revealed the presence of mixed CuFe_5_O_8_ and copper oxide phases, where the formers are rich in Cu^2+^, Fe^2+^, and Fe^3+^ ions. The electron transfer between Cu^2+^, Fe^2+^, and Fe^3+^ led to the high efficiency of the catalytic reaction of the synthesized copper ferrites. Especially for the sample calcined at 600 °C, the apparent kinetic constant (k) for a reduction of 4-nitrophenol was equal to 0.25 min^−1^, illustrating nearly 100% conversion of 4-nitrophenol to 4-aminophenol within less than 9 min. Regarding the N_2_ adsorption/desorption isotherms, the samples calcined at 600 °C have the highest specific Brunauer–Emmett–Teller (BET) surface area (15.93 m^2^ g^−1^) among the others in the series, which may imply the most effective catalytic performance investigated herein. The post-catalytic X-ray diffraction investigation indicated the stability of the prepared catalysts. Furthermore, the chemical stability of the prepared catalysts was confirmed by its reusability in five consecutive cycles.

## 1. Introduction

Nitrophenol, one of the most important organic compounds, has been widely used in components with significant manufacturing industries, such as dyes, pesticides, and herbicides [1]. Nitrophenols have three structural isomers, namely 2-nitrophenol (2-NP), 3-nitrophenol (3-NP), and 4-nitrophenol (4-NP) [2]. Among others, 4-NP has high stability in a regular environment and relatively high toxicity to hazard human health [3]. After a number of technical advancements, a pathway has been proposed to remedy the contamination of these phenolic compounds, e.g., oxidation processes, photocatalysis, UV irradiation, microwave radiation, electrocatalysis, and the use of noble-metal catalysts [4,5], all of which require state-of-the-art equipment. On the other hand, it has been proposed that a more cost-effective and straightforward pathway to reduce the toxicity of 4-NP is the chemical reduction to the less toxic compound, 4-aminophenol (4-AP) [6,7].

For the removal and conversion of 4-NP to the relative environmental benign 4-AP, Liu et al. prepared a novel ethylenediamine rosin-based resin as an adsorbent material to remove many types of phenol compounds from aqueous solutions. They showed that the highest adsorption efficiency of the resin was found in the case of removing 4-NP [8]. Tan et al. synthesized magnetic imprinted porous foams that achieved the selective identification and physical removal of 4-NP from the aqueous environment [9]. The porosity of the foams was optimized by the addition of cellulose nanocrystals (CNCs). Other techniques for the conversion of 4-NP to 4-AP include the photocatalytic pathway [10], the Fenton effect method [11,12], and the electrochemical methods [13,14].

The catalytic reduction of 4-NP into 4-AP is often regarded as one of the most common, cost-effective, and environmentally friendly methods [15] with the presence of reducing agents, such as NaBH_4_ [16,17]. Reduction reactions using NaBH_4_ have been known to accelerate by the addition of proper heterogeneous catalysts. It has been reported that some oxide groups namely CuO, Co_3_O_4_, Fe_2_O_3_, and NiO could enhance the electron transfer process desired for the reduction reaction, while TiO_2_, V_2_O_5_ Cr_2_O_3_, MnO_2_, and ZnO were inactive [18]. This renders oxide systems containing both Cu and Fe ions subjected to extensive study for the emerging effective catalysts. Several studies have proved that copper ferrite possesses the highest catalytic performance among all metallurgical ferrites due to the effectivity of intervalence electromagnetic transfer between Cu^+^, Cu^2+^, Fe^2+^, and Fe^3+^ ion pairs occupied in the octahedral sites [18,19,20].

Important catalytic roles of copper ferrite are illustrated in the methanol decomposition towards CO and H_2_ production and in the conversion of CO to CO_2_ [21,22]. In addition, copper ferrite particles were employed as the main catalyst for the C-O coupling in the Ullmann reaction, which involves various aryl halides and phenols [23]. Copper ferrite is also utilized as a heterogeneous catalyst in the photosensitive Fenton process due to its narrow band gap, good response to visible light, high photochemical stability, and low production cost [24]. The Fenton catalytic activity for the degradation of organic pollutants using soft magnetic copper ferrite and SiO_2_ nanofibrous membranes has been reached the degradation rate of 96% within 20 min [25]. Green and chemically-engineered copper ferrite, namely CuFe_2_O_4_, has been found to be photoactive and is able to aid the degradation of malachite green under sunlight [26,27]. Furthermore, copper ferrite has recently been proven to be efficient for catalytic ozonation of surfactant-containing simulated laundry wastewater [28]. Other examples of copper ferrite-based materials for the reduction of 4-NP include Cu/CuFe_2_O_4_ nanocomposites [3] and Cu_2_O/Fe_3_O_4_@C/Cu nanocomposites [29]. Interestingly, copper ferrites in the form of CuFe_5_O_8_ have also been reported for catalytic applications [30,31]. Cu-Fe-O systems, including CuFe_5_O_8_, generally have sizeable magnetic properties, which open a pathway for creating reusable magnetic-based catalysts, which are capable of separating organic pollutants from wastewater [32].

As a form of copper ferrite, CuFe_5_O_8_ has been long believed to exist and possess spinel structures with variations in ion distributions among the tetrahedral and octahedral sites. Metastable CuFe_5_O_8_ nanocubes have been proven to combat implanted-related infections, as they act as Fenton-like reaction catalysts, whose activity can be regulated by pH and H_2_O_2_ concentration [31]. The peak fitting results in our X-ray absorption (XAS) measurements, which indicate that all samples contain Cu^2+^, Fe^2+^, and Fe^3+^, demonstrate the metastable state of CuFe_5_O_8_, in agreement with the report by Gorter [33]. In this article, we report on the synthesis of copper ferrite in the form of CuFe_5_O_8_ and systematically investigate its structural characteristics and local structure in detail. A series of CuFe_5_O_8_ catalysts has been prepared via the co-precipitation method. Furthermore, the catalytic activities of the synthesized materials are assessed by the reduction of 4-NP to 4-AP. One of the classes of our synthesized particles has shown excellent catalytic activity toward the reduction of 4-NP by virtue of its relatively high surface area. Above all, the involvement of an efficient co-precipitation method leading to magnetized chemically-active particles will pave a new path in the production of sustainable, eco-friendly, low-cost, and nontoxic catalysts with high catalytic activity.

## 2. Results and Discussion

**Figure 1:**
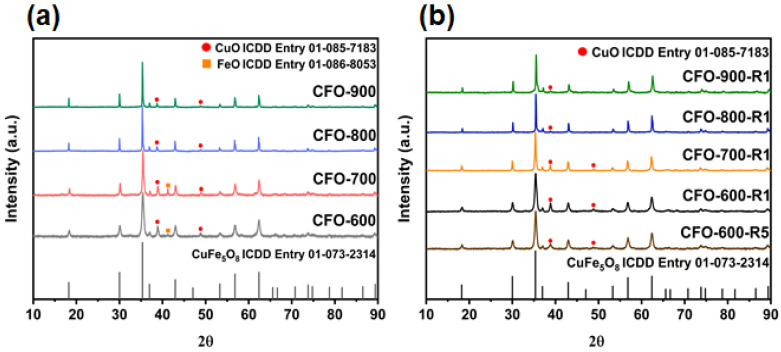
XRD patterns of **CFO-600**, **CFO-700**, **CFO-800**, and **CFO-900** compared to the standard compounds for the (**a**) original samples and (**b**) post-catalysis samples.

The devised co-precipitation route led to final chemical products were later determined to be CuFe_5_O_8_/CuO/FeO solid mixtures. The obtained samples were named after the corresponding calcination temperatures of 600 °C, 700 °C, 800 °C, and 900 °C as **CFO-600**, **CFO-700**, **CFO-800**, and **CFO-900**, respectively. The data from X-ray diffraction (XRD) phase analysis and ICDD standard (Figure 1a) showed that the main phases in the freshly prepared **CFO-600**, **CFO-700**, **CFO-800**, and **CFO-900** are mostly CuFe_5_O_8_ (ICDD entry 96-153-8388), which has the *Fd-3m* space group. Past studies on Cu-Fe-O systems mostly focused on the cuprospinel phase (Cu_z_Fe_2−z_O_4_), which occurred in two forms, i.e., the cubic (space group *Fd-3m*) or tetragonal (*I*4_1_/*amd*), in which the former structure is often associated with the structure of copper ferrites. The space group *Fd-3m* represents the structure of a normal spinel AB_2_O_4_, where the oxygen ions form a cubic close-packed structure, in which half of the octahedral interstices are occupied by trivalent B^3+^ cations, and 1/8 of the tetrahedral interstices are occupied by divalent A^2+^ cations [34]. The appearance structure of copper ferrites depends on the compositions of copper and iron in the solid solution as well as calcination temperatures [35,36]. The structure of CuFe_5_O_8_ are reported as (1) a single compound, (2) a solid solution of Cu^II^Fe_2_O_4_ and Fe^II^Fe_2_O_4_, (3) a mixture between CuFe_2_O_4_ and Fe_2_O_3_, and (4) the ionic distributions of Cu^I^_x_Fe^III^_1−x_[Fe^III^_x_(Cu_0.5−x_Fe_0.5−x_)Fe^III^_1+x_]O_4_ or Cu^II^_y_Fe^III^_1−y_[Fe^II^_y_(Cu_0.5−y_Fe_0.5−y_)Fe^III^_1+y_]O_4_ [33].

In our X-ray diffraction study, the characteristic diffraction peaks were found at 12.21°, 19.99°, 23.50°, 28.40°, 35.04°, 37.17°, 40.60°, and 56.20°. By noting the width of these characteristic peaks, it can be observed that the average crystallite size of the CuFe_5_O_8_ particles increases with higher calcination temperatures. The crystallite sizes of all samples are quantified based on an analysis using Scherrer equation, where the XRD peak widths (*B*) are inversely proportional to crystallite sizes (*L*) as

(1) B(2θ)=Kλ/Lcos(θ)

These data are summarized in Table 1. The trend of increasing crystallite size of copper ferrite samples with higher calcination temperature has been recently observed in the work by Lopez-Ramon et al. [36]. Monoclinic copper oxide (matched with ICDD entry 96-901-5888, showed with *) was found in all samples. The impurity phases slightly decrease with higher calcination temperatures. The element compositions, shown in weight percent, and the crystalline phase compositions are also displayed in Table 1. Controlling calcination temperature can usually change the composition of compounds due to the transformation of the crystalline phases. In our case, when the sample was treated at 800–900 °C, larger amounts of CuO within the solid was converted into CuFe_5_O_8_. Data in Table 1 suggest that due to the transformation kinetics, high activation energy is required for the transformation from CuO/FeO to CuFe_5_O_8_ to compensate for oxygen insertion into the CuFe_5_O_8_ structure and the breaking of metal–oxygen bonds in CuO and FeO. Furthermore, the crystallite size of the CuFe_5_O_8_ particles also gradually increases with increasing calcination temperature due to the increase in the aggregation of particles. It is known that particles clumping at a higher calcination temperature tends to lower the chemical characteristics, rendering it difficult for the particles to be used as catalysts. Hence, it is anticipated that the sample **CFO-600** may be the most chemically active in converting 4-NP to 4-AP since it possesses the smallest crystallite size while acquiring a just-sufficient amount of active Cu^2+^, Fe^2+^, and Fe^3+^ ions in the chemical mixture CuFe_5_O_8_/CuO/FeO. This point will be examined and discussed later in the sections on BET-surface investigation and the chemical activity study.

Figure 1b shows the XRD phase analysis of samples after the experiments, where the prepared CuFe_5_O_8_ particles have been served as catalysts for the reduction of 4-NP to 4-AP, where the hydrolysis of NaBH_4_ produces four molecules of hydrogen that lead to the reduction of 4-NP. This figure will be discussed in a later paragraph.

**Figure 2:**
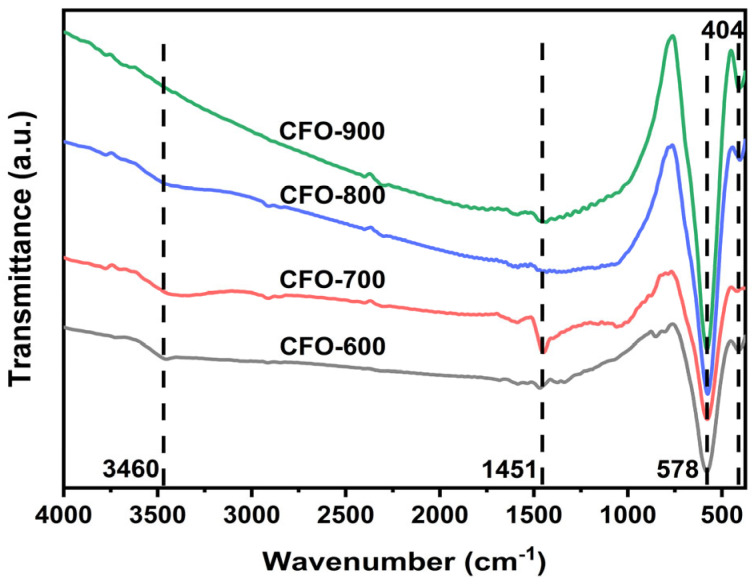
FTIR spectra of the samples calcined at 600 °C, 700 °C, 800 °C, and 900 °C.

Spectra from Fourier transform infrared spectroscopy (FTIR) of all the calcined samples are illustrated in Figure 2. It was found that the O-H peak around 3460 cm^−1^ became less intense as the calcination increased due to the lower water/moisture absorption [37]. The peaks at 578 and 404 cm^−1^ were found in all the samples, which were attributed to M-O bond symmetrical stretching vibrations [38]. When closely examining the peak patterns around 1400 to 1600 cm^−1^, it was noticed that the peak patterns of **CFO-800** and **CFO-900** were similar, indicating similar lattice motion in response to the infrared. Since it was found in the XRD analysis (Table 1) that these two samples have similar crystalline phase composition, the FTIR spectra for **CFO-800** and **CFO-900** thus further confirmed the XRD findings.

**Figure 3:**
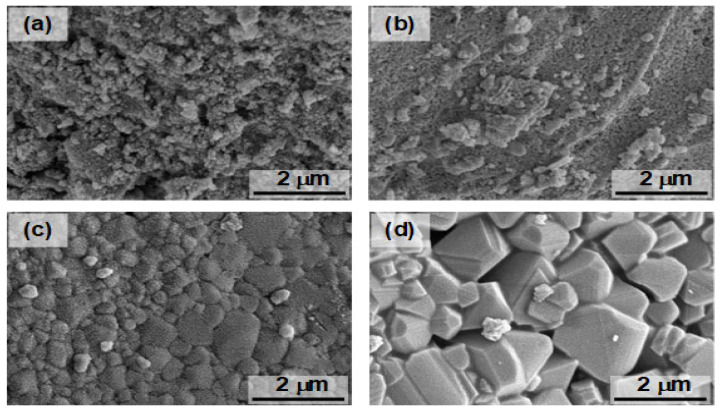
SEM images showing the morphology of the prepared samples obtained from (**a**) 600 °C, (**b**) 700 °C, (**c**) 800 °C, and (**d**) 900 °C calcination temperatures.

**Figure 4:**
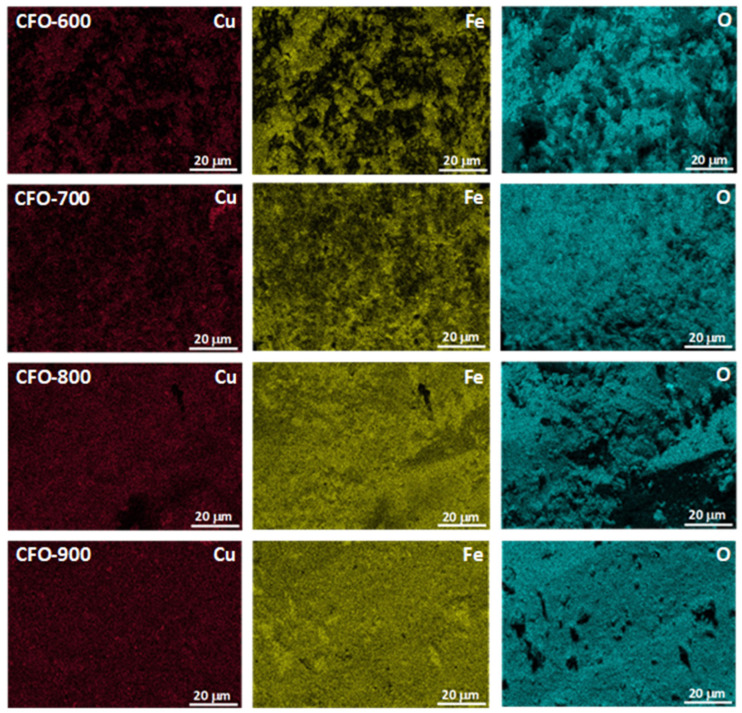
EDS mapping of the prepared samples obtained from various calcination temperatures.

The scanning electron microscopy (SEM) study (Figure 3) illustrates the morphologies of the samples calcined at different temperatures. The sample calcined at 600 °C consisted of spherical particles. As the calcination temperatures increased, the morphology was changed to irregular blocky particles agglomerated together. Smaller particles were found in the **CFO-600** sample, while larger particles due to aggregation were found in the **CFO-700**, **CFO-800**, and **CFO-900** samples. This is consistent with results from the XRD analysis on the crystallite sizes, where high intensities and narrow peaks due to larger particles were observed in XRD patterns of the samples with higher calcination temperatures. The uniformity of the elemental composition of the synthesized samples, after the calcination step, was evaluated using the energy dispersive spectrometer (EDS) element mapping analysis (Figure 4). The EDS demonstrated the homogeneous distribution of Cu, Fe, and O elements in the fabricated copper ferrites in all samples prepared across the calcination temperatures of 600 °C, 700 °C, 800 °C, and 900 °C investigated in this work. The elements of copper (Cu), iron (Fe), and oxygen (O) are consistently spread around whole samples, and no other elements were present in the composition of the prepared particles.

**Figure 5:**
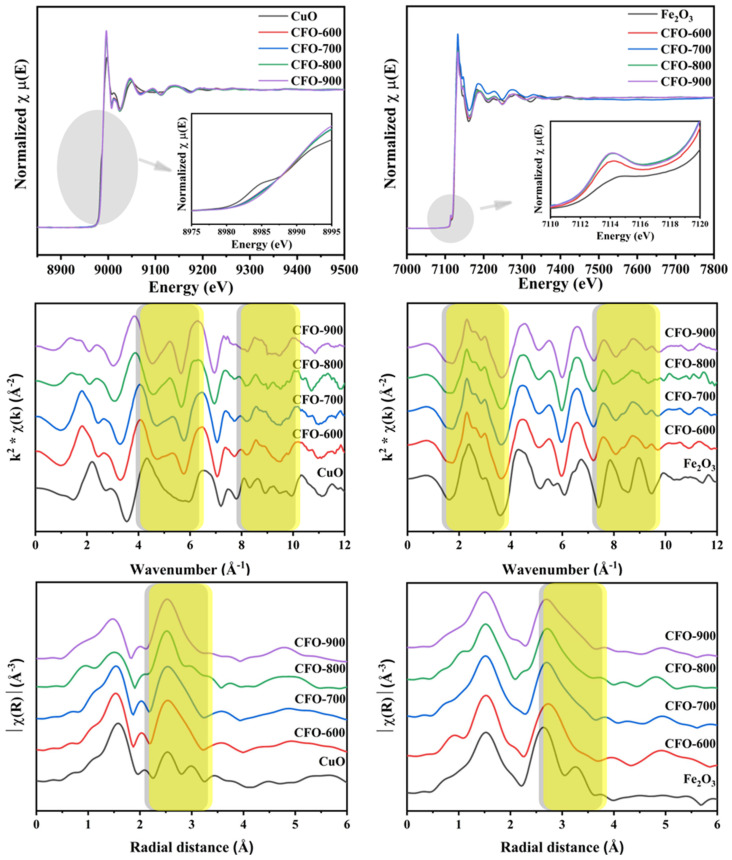
Cu K-edge (**left column**) and Fe K-edge (**right column**) XANES and EXAFS spectra of the samples obtained at different calcination temperatures compared to the CuO and Fe_2_O_3_ standard compounds. Note that, the yellow rectangular highlights the region that shows the difference local structure of the obtained copper ferrites and the reference single-oxide standards.

In order to understand the valency states and the local environments of the metal ions within the samples, an X-ray absorption spectroscopy (XAS) measurement was performed at Cu K-edge and Fe K-edge energies (Figure 5). The left column illustrates the Cu K-edge X-ray absorption near edge structure (XANES) spectra plotted in energy and the corresponding extended X-ray absorption fine structure (EXAFS) spectra of the samples plotted in *k*, and R spaces, while the right column shows the Fe K-edge XANES and EXAFS spectra. When considering the edge positions of XANES spectra in the copper ferrite samples obtained at different calcination temperatures and the CuO standard, it was observed that the edge positions of copper for all the prepared copper ferrite samples and CuO were similar. This suggested that the copper ions in all the copper ferrite samples possessed a +2-oxidation state. However, the XANES spectral features of the copper ferrite samples were significantly different from the CuO standard, indicating the formation of the mixed-metal oxide framework in the samples. Moreover, the pre-edge peak was not noticed in any copper ferrite samples. For the Fe K-edge XANES spectra, the edge positions of FE K-edge in copper ferrite samples were similar to the Fe_2_O_3_ standard. This implied that the iron ions possessed mixed valency states of +2 and +3. The pre-edge peak in Fe K-edge XANES spectra of the copper ferrite samples was more prominent than the pre-edge peak of the Fe_2_O_3_ standard. The Fe K-edge XANES features differed notably from the Fe_2_O_3_, where the sample calcined at 700 °C had mostly deviated. This confirmed the XRD results that the calcination temperature of 700 °C gave the highest content of the CuFe_5_O_8_ phase. The evidence of all samples containing Cu^2+^, Fe^2+^, and Fe^3+^ demonstrates the metastable state of CuFe_5_O_8_, in agreement with the report by Gorter [33].

As observed from the Cu K-edge EXAFS spectra in *k* and R spaces, the first coordination shell of the copper ferrite samples and the CuO standard were similar, as the first shell corresponded to the Cu-O environment. However, the EXAFS spectra of the copper ferrite samples and the standard CuO were prominently different at *k* around 5–5.5 Å^−1^ and 8–10 Å^−1^ (R around 2.2–3.5 Å and 4–6 Å), referring to the different second and third coordination shells. This was due to the different scattering paths of the electron in the copper ferrite and copper oxide frameworks.

**Figure 6:**
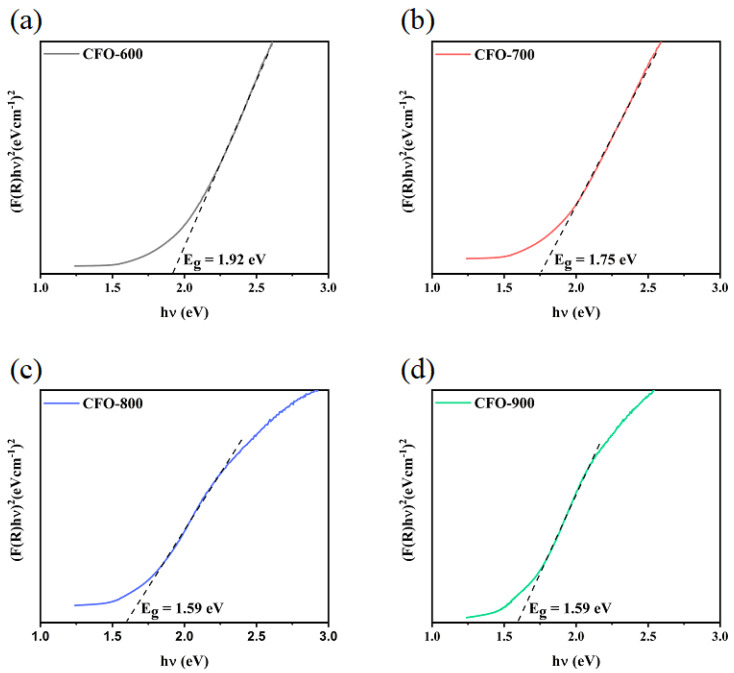
(**a**–**d**) Reflectance spectrum of samples obtained at different calcination temperatures. The spectrum was plotted against photon energy. The associated Tau plot for the determination of the band gap energy was also shown.

Kubelka–Munk transformed reflectance spectra plots as functions of photon energy of **CFO-600**, **CFO-700**, **CFO-800**, and **CFO-900** are illustrated in Figure 6. The band structure of copper ferrite was found through Tauc’s relation [39].

(2) $$\left(\alpha h\nu {)}^{2}=C(h\nu -E_{g}\right)$$

where *hv = hc*/*λ*; Planck’s constant *h* = 4.136 × 10^−15^ eV s and the velocity of light *c* = 3 × 10^17^ nm s^−1^; *hc* = 1240 eV nm; *λ* denotes absorbance wavelength; *α* denotes an absorption coefficient; *E_g_* denotes the energy of the optical band gap; and *F*(*R*) denotes a proportionality constant. The plotting of (*αhv*)^2^ vs. the photon energy (*hv*) gives a straight line in a certain energy range. The extrapolation of this line intercepts the energy axis at the value of the direct optical energy band gap (*E_g_*). The band gap energy of all samples was found to be in the range of 1.59–1.92 eV. The results were consistent with the direct band gap values of 1.9–2.04 eV for the spinel copper ferrite (CuFe_2_O_4_) reported in the literature [40,41,42]. The reduction in the *E_g_* value for the chemical mixture was associated with lower possible photon energy and implies transition across the gap. Therefore, copper ferrite samples with higher calcination temperatures possess fewer active electrons that may facilitate improvement in the reduction of 4-NP to 4-AP, as compared to the active electrons in the **CFO-600** sample.

**Figure 7:**
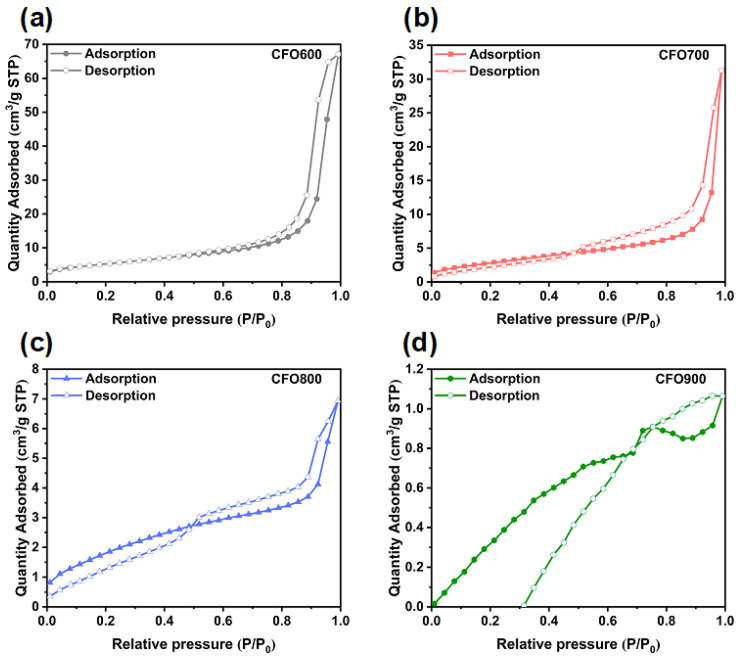
Nitrogen adsorption/desorption isotherm for samples obtained at calcination temperatures of (**a**) 600 °C, (**b**) 700 °C, (**c**) 800 °C, and (**d**) 900 °C.

The surface analysis using the Brunauer–Emmett–Teller (BET) equation (S_BET_) illustrated that each sample had a correlation between the surface areas to calcination temperatures. The measured surface areas are displayed in Table 2, while the nitrogen adsorption/desorption isotherms are shown in Figure 7. It can be seen that the surface area of **CFO-600** is the highest, with a value of 15.93 m^2^/g. The decreasing surface areas were observed in samples with higher calcination temperatures, i.e., 4.67, 0.82, and 0.08 m^2^/g for **CFO-700**, **CFO-800**, and **CFO-900**, respectively. Interestingly, maximum surface areas and pore volumes were exhibited at 600 °C calcination temperature, as shown in Figure 7. Moreover, the **CFO-600** and **CFO-700** materials exhibited type IV isotherm (mesopore), while the **CF-800** and **CF-900** showed type II isotherm (macropore). The underlying cause of this observation is most likely due to the initial decompositions of Cu(NO_3_)_2_ to CuO and Fe(NO_3_)_3_ to FeO, as implied by X-ray diffraction results in Figure 1a. The removal of initial chemisorbed water led to more pores being created. When increasing the temperature from 700 °C to 900 °C, primary CuFe_5_O_8_ started to aggregate and form larger secondary particles, as revealed in Figure 1 via the XRD analyses. The drop in the surface area and pore volume possibly indicates that tiny intraparticle voids or micropores are eliminated during the higher-temperature calcination. This is consistent with the XRD results, which demonstrate higher XRD intensities and larger average crystallite sizes with increasing calcination temperatures. Furthermore, nitrogen (N_2_) gas adsorption/desorption isotherms of all the samples can be classified as Type II as a non-porous substrate. This means that the chemical activity of reduction mostly occurs at the surface of the samples. It should be noted that with the current cutting-edge technological advancements in developing diverse structures and unique features of metal oxides, they are among the functional materials that are suitable for catalytic study. However, many oxides possess challenges since their particles tend to clump and aggregate, lowering their chemical characteristics and reducing the prospect of particle recovery from reaction mixture and reuse. Mesoporous oxides, such as **CFO-600** sample with the largest specific BET surface area in the series, are therefore in favor as a chemical candidate for the conversion of 4-NP to 4-AP. Upon further investigation into chemical activities, it will be tabulated and shown that **CFO-600** samples have comparable catalytic activities comparable to those complex structures, such as dispersive metal oxide nanoparticles on supporting substrates (carbon compounds, zeolites, among others). The present surface analysis data indicated that CuFe_5_O_8_ produced at 600 °C calcination temperature has the most likelihood of having the highest catalytic activity due to its highest specific surface area. These characteristics promote the electron transfer mechanism during the catalytic reaction of 4-NP in the vicinity of the surface acidic sites of Cu^2+^, Fe^2+^, and Fe^3+^ ions.

**Figure 8:**
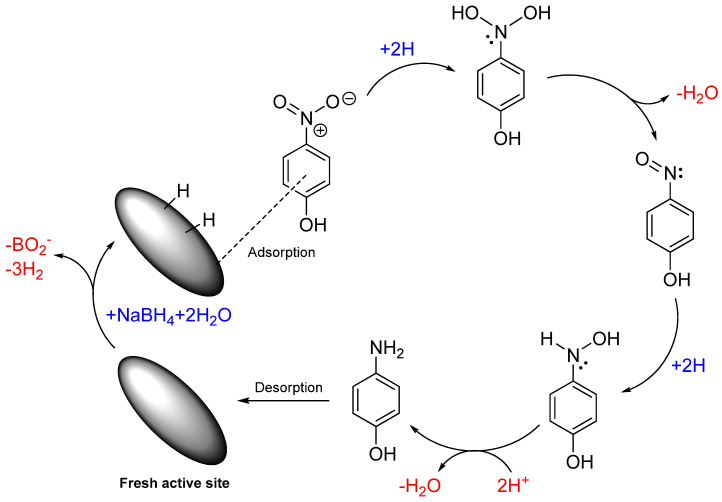
The probable mechanism of the reduction of 4-NP by CuFe_5_O_8_ materials.

CuFe_5_O_8_ serves as a catalyst for the reduction of 4-NP to 4-AP. The borohydride reaction with a metallic surface has become a topic of research in recent years due to the capacity of borohydrides in generating hydrogen [43]. Here, we postulate the reaction mechanism of 4-NP reduction based on the Langmuir–Hinshelwood model and depict the mechanism as shown in Figure 8 [44]. There are a number of reports on the same probable mechanism over other catalysts, confirming our assumptions [45,46,47]. The chemical route by which 4-AP is obtained involves direct hydrogenation of 4-NP using sodium borohydride, which is generally a milder agent in aqueous medium. The chemical process starts with self-hydrolysis of NaBH_4_ as

NaBH_4(aq)_ +2H_2_O → 4H_2(g)_ + NaBO_2(aq)_

The hydrolysis of NaBH_4_ produces four molecules of hydrogen that lead to the reduction of 4-NP. The produced hydrogen is then adsorbed on the surface of the catalyst following by a hydrogenation reaction. These steps are repeated three times to reach the full reduction of the nitro group to the amine group. The reduction process consumes six proton (H^+^) and involves a six-electron reduction of 4-NP in the presence of catalyst and an excess amount of NaBH_4_. In the reduction of 4-NP, the consumption of the produced hydrogen, from the self-hydrolysis of NaBH_4_, shifts the reaction forward. This further enhances the hydrolysis of NaBH_4_. NaBH_4_ therefore serves as the reducing agent in the aqueous solutions [48,49,50]. CuFe_5_O_8_ contains acidic (Cu^2+^, Fe^2+^, Fe^3+^) and basic (O^2−^) sites. The acidic sites on the surface of the metal oxide promote the breakage of the N-O bond in the intermediate phenylhydroxylamine while the basic sites tend to attract the dissociated hydrogen of NaBH_4_. Therefore, the presence of acidic/basic sites on catalysts helps speed up the reaction. The overall hydrogenation can be expressed as a six-electron process:

C_6_H_5_NO_3_ + 6H^+^ + 6e^−^ → C_6_H_7_NO + 2H_2_O

The ultraviolet–visible spectroscopy (UV–Vis) absorption spectrophotometer was operated to evaluate the reactive performance of CuFe_5_O_8_ in the reaction between 4-nitrophenol and NaBH_4_. The solution of 4-nitrophenol and NaBH_4_ has a luminous yellow color, which was observed as the absorption peak at 400 nm [3].

**Figure 9:**
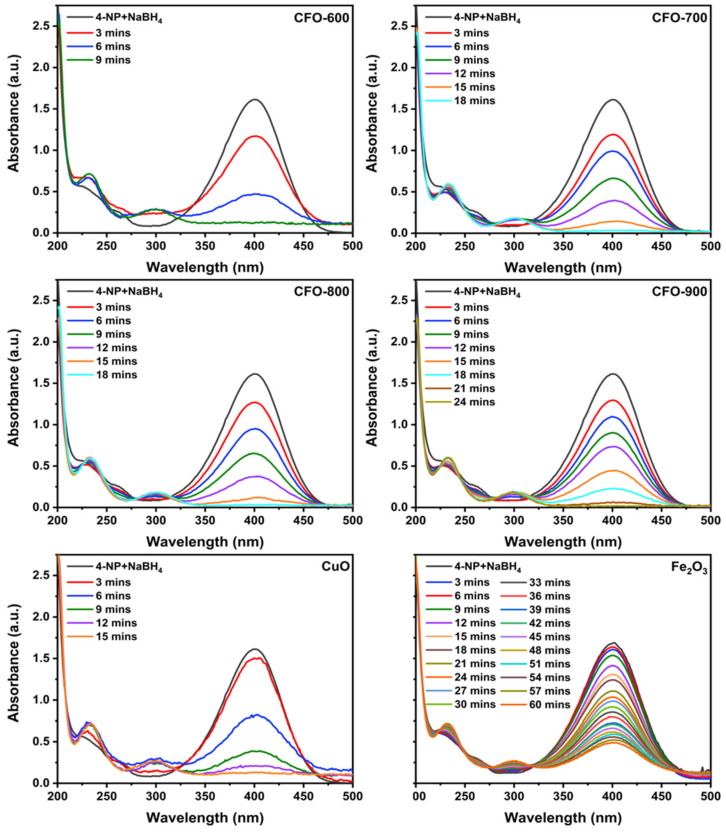
UV–Vis spectra of the reaction mixtures for the reduction of 4-NP to 4-AP using the copper ferrite samples obtained at different calcination temperatures and NaBH_4_.

**Figure 10:**
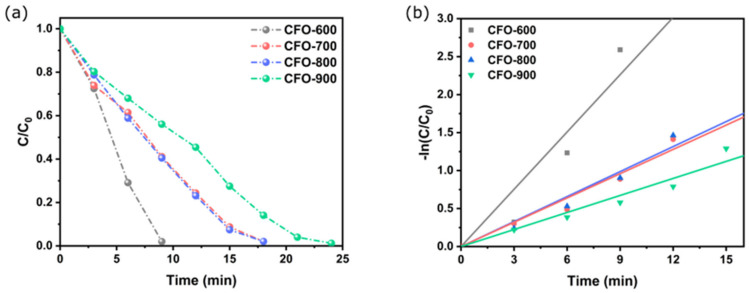
(**a**) The plot of C/C_0_ against time and (**b**) the plot of ln(C/C_0_) against time for the reduction of 4-NP to 4-AP using the prepared copper ferrite samples obtained from various calcination temperatures.

**Figure 11:**
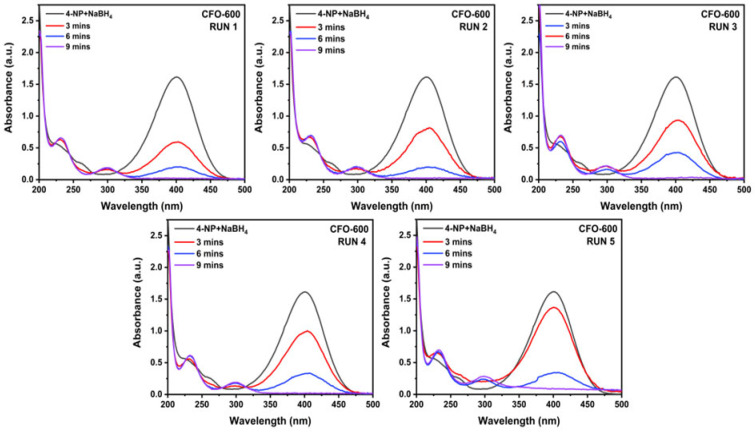
Reaction times of the complete reduction of 4-NP to 4-AP by NaBH_4_ using the new and reused copper ferrite sample, **CFO-600** up to 5 times.

After instilling copper ferrite in the solution, 4-aminophenol was observed as an appearance peak around 300 nm [51]. The UV–Vis absorption spectra of the copper ferrite samples and NaBH_4_ were recorded every minute (Figure 9). The rate of disappearance of 4-nitrophenol was found to be the highest in the presence of **CFO-600**, as the peak at 400 nm was totally diminished within 9 min. The complete reaction times were extended to 9, 18, and 15 min for **CFO-700**, **CFO-800**, and **CFO-900**, respectively (Figure 10a). In order to calculate the rate constant, the linear graphs of −ln(C/C_0_) against time were plotted (Figure 10b), and the gradients (rate constants) of the linear lines were calculated. The rate constants for **CFO-600**, **CFO-700**, **CFO-800**, and **CFO-900** were 0.29, 0.20, 0.21, and 0.17 min^−1^, respectively. The pseudo-first-order reaction rate constants are summarized in Table 3, along with the R^2^ values that reflect the goodness of the fittings. The table demonstrates that all catalysts have good catalytic activity for the reduction reaction of 4-NP. The order of the reactivities is the following: **CFO-600** > **CFO-700**~**CFO-800** > **CFO-900**. This is consistent with the study on nitrogen adsorption/desorption isotherms (Figure 8), where it has been argued that the trend of decreasing surface areas with higher calcination temperatures is observed. However, the degree of reactivities is inversely dependent on the amount of purity CuFe_5_O_8_ phases, as seen in the X-ray analysis in Table 1. Both the nitrogen adsorption/desorption isotherm and X-ray results implied that the reaction extent in this work mainly depended on the catalyst surfaces, not the purity of the chemical species in the prepared samples. As comparative studies, the UV–Vis absorption experiments were carried out for commercial CuO and Fe_2_O_3_ as catalysts. It was found that in the same reaction conditions, the catalytic activities for the reduction of 4-NP to 4-AP using NaBH_4_ are in the following order: **CFO-600** > CuO > Fe_2_O_3_. The recyclability of the catalysts was also investigated by performing the reaction using the catalysts separated from the previous reaction for up to five cycles of reduction, filtration, washing, and drying. The study revealed that the prepared catalysts were still active at up to five revolutions (Figure 11).

Post-catalysis X-ray characterization of all samples has been performed, and the results have been shown in Figure 1 and Table 1. The fresh and post-catalytic samples were characterized by X-ray diffraction in order to analyze the crystal phases present and the crystallinity of each phase. As seen in Figure 1b, almost all of the peaks in the pattern after the reaction could be indexed as CuFe_5_O_8_. With investigated accompanying crystalline phase compositions (Table 1), it could be observed that the chemical content of the as-prepared **CFO** samples has noticeably changed. This is due to the formation of a single phase of cubic CuFe_5_O_8_, with a *Fd-3m* space group having occurred as the 4-NP reduction reactions progresses. Similar formation toward a single phase after the catalytic reactions of copper ferrites has been reported by other researchers, who investigated catalytic activities of copper ferrite in the oxidation of carbamazepine [52]. The XRD pattern of the post-catalytic sample shows that the peaks that correspond to the CuFe_5_O_8_ structure are alike observed for the as-prepared **CFO** samples. This indicates the retention of the crystalline structure features throughout five consecutive catalytic cycles. The above results therefore suggest the robust nature of the catalyst and its efficiency for the reduction of 4-NP into 4-AP without much reduction in the activity.

Several other catalysts containing either Cu or Fe ions were reported for the reduction of 4-NP to 4-AP. Some of them are listed in Table 4 [46,53,54,55,56,57]. These materials offer high efficiency and high recyclability. However, CuFe_5_O_8_ also exhibits a short reaction time compared to the catalyst TiO_2_/CoFe_2_O_4_ [53]. Fe_3_O_4_/P (GMA-DVB)/PAMAM/Au suffers from high production costs due to the incorporation of the precious noble metal in their structures [54]. Also, this material showed an efficiency of 78.7%, while CuFe_5_O_8_ offers a complete conversion of 4-NP to 4-AP. The production of Cu_2_O/Cu-MOF/rGO required the preparation of graphene oxide from natural graphite using a modified Hummer’s method [55], while for the current CuFe_5_O_8_ production, only a one-step co-precipitation method was needed. For the use of natural Fe_2_O_3_ for the reduction of 4-NP to 4-AP, it was found that the efficiency of 99% was found within the reaction time of 4 min [46]. However, in our comparative study, as reported in Figure 10, it was found that in the same reaction conditions as ours, natural Fe_2_O_3_ did not reach the efficiency of 99% even though the reaction time lasted 60 min. The synthesis procedure that CuFe_5_O_8_ requires can be considered simple compared to other systems that require several steps and days for producing a catalyst. Though the reaction time for achieving 100% efficiency is a little longer than those of other systems (in Table 4), it is within the order of minutes. The difference in the reaction time could be attributed to the number of catalysts and reaction conditions set for different systems in Table 4. Further comparisons of our CuFe_5_O_8_ calcined at 600 °C, Cu-based (CuO) samples, and Fe-based (Fe_2_O_3_) samples are shown in Table 5. The data on the Cu-based and Fe-based samples are taken from the literature. It can be seen that the synthesized CuFe_5_O_8_ has comparable reaction rate constants to those of CuO and Fe_2_O_3_. The efficiency in the conversion of 4-NP to 4-AP in this group of oxides correlates with their respective band gap energy values [58]. The band gap energy of CuO and Fe_2_O_3_ have been reported to be 1.7 eV and 2.2 eV, while our **CFO-600** samples possess the band gap energy value of 1.92 eV. It has been reported that the lower band gap, in the case of the oxides in this group (CuO and Fe_2_O_3_), facilitates faster electron transfer. This strongly suggests that our **CFO-600** samples, abundant in Cu^2+^, Fe^2+^, and Fe^3+^ and having comparable band gap energy, can be considered to possess good chemical activities in the conversion of 4-NP to 4-AP.

## 3. Materials and Methods

### 3.1. Materials Preparation

The synthesis of metal ferrites in this work was based on the co-precipitation method. Initially, the stoichiometric ratio of metal ions to ferrite was kept at M^2+^:Fe^3+^ equal to 1:2. Initially, the solution was prepared by dissolving 5 mmol of divalent ions from copper nitrate (Cu(NO_3_)_2_ (% purity) with 10 mmol of trivalent ions from iron(III) nitrate (Fe(NO_3_)_3_) (% purity) in 50 mL of deionized water. Afterward, the solution was continuously stirred until homogeneity was observed. An amount of 25 mL sodium hydroxide (NaOH) (% purity) was then gradually dropped into the solution. The calculated ionic ratio of Cu/Fe/OH is 1:2:8. The complete precipitation was assumed after stirring for 30 min. The obtained residue was filtered and washed with deionized water and later ethanol. The drying process was carried out at 60 °C for 24 h in a hot air oven. Finally, the samples were calcinated at 600 °C, 700 °C, 800 °C, and 900 °C for 6 h in the ambient air. The final products were later determined to be CuFe_5_O_8_/CuO/FeO mixtures. The samples were named after the corresponding calcination temperatures as **CFO-600**, **CFO-700**, **CFO-800**, and **CFO-900**.

### 3.2. Catalytic Testing

The preparation step for catalyst testing was presented as follows: 0.1 mM of 4-nitrophenol (C_6_H_5_NO_3_, MW 139.11 g/mol) was prepared, and then, 60 mM of sodium borohydride (NaBH_4_, MW 37.83 g/mol) was weighed. An amount of 3 mL of 4-nitrophenol and fresh 0.5 mL of NaBH_4_ solution were mixed and added with 5 mg of CuFe_5_O_8_ particles. The catalyst property was traced, using UV–Visible spectroscopy, by monitoring the color of 4-NP (yellow color), which gradually disappeared to a colorless solution.

The catalytic activity investigation was conducted in cycles. After the initial 4-NP reduction process was finished, CuFe_5_O_8_ was separated from the solution using a magnet, which was placed outside the beaker to attract the magnetic CuFe_5_O_8_ particles. The solution was then discarded while the remaining magnetic particles were collected. The particles were then rinsed with deionized water and ethanol before being dried in an air oven at 60 °C for 15 min. Afterward, the catalytic testing of the next cycle was repeated. A total number of 5 cycles were performed in this experiment.

### 3.3. Materials Characterization

#### 3.3.1. X-Ray Diffraction (XRD)

The crystallographic structure investigation of copper was acquired by X-ray diffraction (XRD) technique at BL1.1W Siam Photon Laboratory, Synchrotron Light Research Institute (Public Organization), Nakhon Ratchasima, Thailand, using monochromatic synchrotron X-ray with a wavelength of 1.033200 Å at room temperature within 2Ɵ angular range of 6.95°–67.08° using 1-dimensional strip detector (Mythen 6K, Dectris®, Baden-Dättwil, Switzerland) for XRD data collection with an exposure time of 800 s.

#### 3.3.2. Scanning Electron Microscopy/Energy Dispersive X-Ray Spectroscopy (SEM/EDS)

For this investigation, the sample morphology and particle size were analyzed by employing scanning electron microscopy (SEM), model Hitachi-SU3500 (Hitachi High-Technologies Corporation, Tokyo, Japan), operated at an accelerating voltage of 10 kV. The energy dispersive X-ray spectroscopy (EDX) mode was used to perform a rough quantitative elemental analysis of the samples.

#### 3.3.3. X-Ray Absorption Spectroscopy (XAS)

XAS is an invaluable tool for studying chemical and electronic structure of metal oxides. The determination of the oxidation state for metals is carried out with a feature for detecting X-ray absorption near-edge structure (XANES) in the spectroscopy. On the other hand, extended X-ray absorption fine structure (EXAFS) spectra can, in principle, provide quantitative insights into the distribution of atoms around the metal ion sites. The examination of the oxidation states and the local structures for the copper and iron centers in our synthesized **CFO-600**, **CFO-700**, **CFO-800**, and **CFO-900** were performed using XANES and EXAFS, respectively. Measurements were also performed on CuO and Fe_2_O_3_ reference materials for comparisons. The spectroscopy probed at Cu and Fe K-edge were collected in the transmission mode at Beamline 1.1 W, SLRI, Nakhon Ratchasima, Thailand. The adjustment of the diffracted angle of a pair of Si(111) crystals in a fixed-exit double crystal monochromator (DCM) allowed for the selection of a series of monochromatic X-ray beams. The intensities of both the incident and transmitted X-rays were monitored using an ionized chamber. The energy scan range of −150 eV, −20 eV, 60 eV, 200 eV, and 15 k (EXAFS range) relative to the Fe and Cu absorption edge were used for the measurements around Fe and Cu metal centers, respectively. Specific scan step sizes of 5 eV, 0.2 eV, 1 eV, and 0.05 eV per step for each energy interval were set during scanning. A collection time of 1 s per point was used for XAS scanning. The XANES spectra specifically focused on the energy range between −20 eV and + 60 eV relative to the absorption edge. To minimize the effects of experimental setting to the detected spectra, simultaneous in-line measurements of the samples and the standard Fe foil and Cu foil were performed to calibrate the energy shifts caused by the instrumental set up. The collected data were analyzed using ATHENA software (version 0.9.26).

#### 3.3.4. Ultraviolet–Visible Spectrophotometer (UV–Vis)

The concentration of 4-nitrophenol changing was traced with ultraviolet–visible spectrophotometer model Shimadzu UV-1700 PharmaSpec (Kyoto, Japan) in absorption mode using a wavelength range starting at 500 nm and ending at 300 nm with a scan speed of 800 nm/min.

#### 3.3.5. Brunauer–Emmett–Teller (BET)

The specific surface area was investigated by Brunauer–Emmett–Teller (BET) with 3Flex Version 5.02 of Micromeritics Instrument Corporation (Norcross, GA, USA). The moisture was reduced by the degassing process at 150 °C for 12 h. Then, the N_2_ adsorption/desorption isotherms were measured. The specific BET surface areas were then calculated at the P/P_0_ range from 0.01 to 0.03 using the BET equation.

## 4. Conclusions

Phase compositions of the copper ferrite prepared by co-precipitation and then treated with different calcination temperatures were investigated for probable potentials in 4-NP reductions. The CuFe_5_O_8_ was the main phase of all the samples confirmed by XRD, where the sample calcined at higher temperatures possessed the highest content of the metastable CuFe_5_O_8_. XAS studies revealed the Cu valency state of +2 and Fe valency states of the mixture of +2 and +3. XAS results also confirmed the development of the copper ferrite framework due to the distinct difference in XANES pre-edges, XANES features, *k*-space EXAFS oscillations, and Fourier transform EXAFS radial distribution functions (especially the second and third coordination shells). In investigating the catalytic activities towards the reduction of 4-NP to 4-AP, it was found that the catalytic performances were mainly dependent on the catalyst surface area rather than the purities of cupper ferrite. It has been noted that the sample calcined at 600 °C with the highest specific BET surface area demonstrated the fastest disappearance of the absorption peak of 4-NP within 9 min, though this sample does not possess the highest CuFe_5_O_8_ chemical contents, as found in the X-ray analysis. The recyclability of the prepared catalysts was determined by reusing the catalyst to carry out the reaction. According to the study, it was found that the catalytic activities were relatively stable for up to five cycles of the experiment. Furthermore, the post-catalytic analyses indicate that the catalyst retains its phase throughout the catalytic cycles. The co-precipitation synthesis method for creating CuFe_5_O_8_ particles broadens the scope of a recyclable catalyst for the reduction of 4-NP and can be considered as an economical and eco-friendly step toward sustainable industrial waste reduction.

## Figures and Tables

**Table 1:** Elemental compositions, crystalline phase compositions, and crystallite sizes.

Sample	Elemental Composition (Weight %)			Crystalline Phase Composition (Amount %)						Crystallite Size (nm)
				Before Reaction			After Reaction			
	Cu	Fe	O	CuFe_5_O_8_	CuO	FeO	CuFe_5_O_8_	CuO	FeO	
**CFO-600**	27.08	57.76	15.16	77.10	20.20	2.60	80.0	20.0	0.0	18.48
**CFO-700**	27.86	61.21	10.94	64.80	29.60	5.50	90.50	9.50	0.0	27.10
**CFO-800**	27.86	61.20	26.03	86.90	13.10	0.0	93.00	7.00	0.0	52.42
**CFO-900**	27.08	57.76	15.16	86.20	13.80	0.0	96.50	3.50	0.0	55.07

**Table 2:** Surface areas of the samples calcined at different temperatures.

Sample	Specific BET Surface Area (m^2^ g^−1^)
**CFO-600**	15.93
**CFO-700**	4.67
**CFO-800**	0.82
**CFO-900**	0.08

**Table 3:** Pseudo-first-order reactions of 4-NP reduction to 4-AP for the full conversion (>99%).

Sample	*k* (min^−1^)	R Square
**CFO-600**	0.25	0.96
**CFO-700**	0.11	0.99
**CFO-800**	0.11	0.98
**CFO-900**	0.07	1.00

**Table 4:** Comparative data of the catalytic activities of several catalyst systems from the literature, including one in this current study, on the reduction of 4-NP to 4-AP.

Catalyst	Synthesis	Reaction Conditions	Conversion	Time	Ref
		(4-NP, NaBH_4_, Cat, T)	(%)	(min)	
Fe_2_O_3_	Natural hematite	0.1 mM, 0.5 M, 1 mg, 25 °C	99	4	[46]
TiO_2_/CuFe_2_O_4_	Co-precipitation method	0.05 mmol L^−1^, 0.22 M, Membrane, 25 °C	95	35	[53]
	Chemical impregnation technique in ultrasounds				
Fe_3_O_4_/P(GMA-DVB)/PAMAM/Au	Solvothermal method, 40 °C for 2 h	0.60 mM, 0.10 M, 1.0 gL^−1^ catalyst, 45 °C	78.7	7	[54]
	Polymerization method, 2 h at 90 °C				
	Michel addition and amidation 60 °C for 48 h				
Carbonization of Fe-BDC MOF	Solvothermal DMF and water at 100 °C, 24 h	20 ppm, 0.5 M, 5 mg, 25 °C	100	4	[55]
	Carbonization at 800 C in Ar gas atmosphere				
Cu_2_O/Cu-MOF/rGO	Modified Hummer’s method	0.1 mM, 0.1 M, 1 mg, 25 °C	100	2	[56]
	Solvothermal 110 °C for 36 h				
	70 °C under reflux conditions, 5 h				
CuBDC	Solvothermal 100 °C for 5 h	0.1 mM, 0.1 M, 5 mg, 25 °C	100	2	[57]
CuFe_5_O_8_	Co-precipitation method	0.1 mM, 60 mM, 5 mg, 25 °C	100	9	This work

**Table 5:** Comparative data on the apparent rate constants for Fe_2_O_3_, CuO, and our CuFe_5_O_8_. The data on Fe_2_O_3_ and CuO are taken from the literature.

Catalyst	Synthesis	Reaction Conditions	k	Ref
		(4-NP, NaBH_4_, Cat, T)	(min^−1^)	
Fe_2_O_3_	Natural hematite	0.1 mM, 0.5 M, 1 mg, 25 °C	0.84	[46]
CuO	99%, S. D. Fine (As purchased)	5 mM, 0.05 M, 0.1 g, 25 °C	1.14	[58]
CuFe_5_O_8_	Co-precipitation method	0.1 mM, 60 mM, 5 mg, 25 °C	0.25	[This work]

## Data Availability

The data presented in this study are available on request from the corresponding authors.
